# Pedobarographic analysis of body weight distribution on the lower limbs and balance after ankle arthrodesis with Ilizarov fixation and internal fixation

**DOI:** 10.1186/s12938-018-0608-z

**Published:** 2018-11-26

**Authors:** Piotr Morasiewicz, Grzegorz Konieczny, Maciej Dejnek, Leszek Morasiewicz, Wiktor Urbański, Mirosław Kulej, Szymon Łukasz Dragan, Szymon Feliks Dragan, Łukasz Pawik

**Affiliations:** 1 Department and Clinic of Orthopaedic and Traumatologic Surgery, Wrocław Medical University, ul. Borowska 213, 50-556 Wrocław, Poland; 2 Faculty of Health Sciences and Physical Education, Witelon State University of Applied Sciences, Legnica, Poland; 3 Department of Physiotherapy and Occupational Therapy in Motor Disorders and Dysfunctions, University of Physical Education, Al. IJ Paderewskiego 35, Wroclaw, Poland

**Keywords:** Pedobarography, Body weight distribution, Balance, Ankle arthrodesis, Ilizarov fixation, Internal fixation

## Abstract

**Background:**

A number of various techniques were proposed to stabilized ankle arthrodesis, among them external and internal fixation. Appropriate balance and adequate distribution of lower limb loads determine normal biomechanics of the locomotor system. We hypothesized that various techniques used to stabilize ankle arthrodesis may exert different effects on (1) balance and (2) distribution of lower limb loads.

**Methods:**

Retrospective analysis included 47 patients who underwent ankle arthrodesis with external stabilization with Ilizarov fixator (group 1, n = 21) or internal stabilization with screws (group 2, n = 26) between 2007 and 2015. Balance and distribution of lower limb loads were determined with a pedobarographic platform.

**Results:**

In group 1, average load of the operated and non-operated limb amounted to 48.8% and 51.2%, respectively, and in group subjected to internal stabilization to 48.4% and 51.6%, respectively. Neither the intragroup nor the intergroup differences in the distribution of lower limb loads were statistically significant. Mean length of the center of gravity (COG) path was 137.9 cm for group 1 and 134 cm for group 2, and mean COG area amounted to 7.41 cm^2^ and 6.16 cm^2^, respectively. The latter intergroup difference was statistically significant.

**Conclusions:**

Balance after ankle arthrodesis with Ilizarov fixation is worse than after the same procedure with internal stabilization. Despite correction of ankle deformity, musculoskeletal biomechanics still remains impaired. While ankle fusion with either Ilizarov or internal fixation provide appropriate distribution of lower limb loads, none of these procedures normalize patients’ balance.

## Background

Ankle joint degeneration and deformity may contribute to mobility limitations, pain, static and dynamic musculoskeletal disorders [1–11]. Ankle arthrodesis is a common procedure in patients with severe ankle arthritis [12–26].

A number of various techniques were proposed to achieve ankle arthrodesis, among them external and internal stabilization [14–16, 18, 20, 22, 23, 25, 26]. Outcomes of ankle arthrodesis vary depending on the stabilization technique [14, 22, 24, 26–30].

Restoration of appropriate musculoskeletal biomechanics requires correction of both lower limb axis and length [1, 2, 8–11]. Musculoskeletal function under static (balance, lower limb load distribution) and dynamic conditions can be evaluated using a pedobarographic platform [9–11, 31–33].

Proper function of lower limbs requires both normalization of balance and symmetrical distribution of loads [9–11, 31–36]. Postoperative improvement of lower limb function is a key determinant of satisfactory treatment outcome [12, 13, 37, 38].

An effective technique of ankle arthrodesis should provide both normal distribution of limb loads and appropriate balance; this enables the patient to involve in the activities of daily living and sport activities [9, 10, 34, 39, 40].

In the previous paper authors introduced radiological evaluation of ankle arthrodesis with Ilizarov fixation compared to internal fixation [26]. In this article, we noted that Ilizarov fixation of ankle arthrodesis is associated with lower prevalence of adjacent-joint arthritis and ankle joint misalignment, and with higher fusion rates than after internal fixation [26].

To the best of our knowledge, no other previous study has analyzed balance and distribution of lower limb loads after ankle arthrodesis depending on the type of its stabilization technique, for example Ilizarov fixation versus internal fixation.

The knowledge of balance and distribution of lower limb loads will be helpful in determining which of the techniques used to stabilize ankle arthrodesis provide better biomechanical outcomes. The principal aim of our present study was to verify which technique used to stabilize ankle arthrodesis provides better outcomes in terms of lower limb biomechanics. We hypothesized that various techniques used to stabilize ankle arthrodesis may exert different effects on (1) postoperative balance and (2) distribution of lower limb loads.

## Methods

Retrospective clinical analysis included 55 consecutive patients who underwent ankle arthrodesis with external stabilization with Ilizarov fixator (group 1) or internal stabilization with screws (group 2) at our institution between 2007 and 2015 [26].

Indications to ankle arthrodesis included severe primary or secondary (post-traumatic, neurogenic, rheumatoid, congenital) degenerative-deforming changes of the ankle joint.

The study subjects underwent ankle arthrodesis with either external Ilizarov fixation or internal stabilization with cannulated screws.

Inclusion criteria of the study were: history of ankle arthrodesis with external stabilization with Ilizarov fixator or internal stabilization with screws, more than 24 months elapsed since the end of the treatment, availability of baseline information on the etiology of ankle pathology and demographic data in medical records, availability of postoperative pedobarographic data. The study subjects were excluded from the analysis if they suffered from Charcot neuroarthropathy, multiple joint injuries or bilateral ankle injuries, or were subjected to additional procedures during the surgical intervention [26].

Patients were enrolled based on their medical history, results of physical examination, pedobarographic examination, analysis of pretreatment and post-treatment medical records. All subjects were informed that their enrollment was fully voluntary, and consented for participation in the study, filling-in all necessary questionnaires and processing of their personal data. In the case of underage subjects, written informed consent was sought from their legal guardians. Protocol of the study was approved by the Local Bioethics Committee [26].

A total of 55 patients underwent ankle arthrodesis between 2007 and 2015. This group included 24 patients subjected to external fixation with Ilizarov device and 31 in whom internal stabilization with screws was used. The pedobarographic tests were done after surgeries. All participants had the post-operation pedobarographic evaluation more than 24 months elapsed since the end of the treatment. One patient from the Ilizarov group (4%) was lost to follow-up before 2 years, another one (4%) had incomplete medical records, and one person (4%) was excluded due to presence of bilateral ankle injuries. As a result, 21 patients subjected to external Ilizarov stabilization were eventually eligible for the analysis. These 21 patients (7 women and 21 men) were followed-up for 45 months on average (range 24–108 months). Two patients from the internal stabilization group (6%) were lost to follow-up before 24 months, 2 (6%) were excluded from the analysis due to incomplete medical documentation, and 1 (3%) due to presence of neuropathic arthropathy, bilateral ankle injuries and performing additional ankle procedures at the time of arthrodesis. Therefore, our analysis included a total of 26 patients (9 women and 17 men) from the internal stabilization group. Mean follow-up for these 26 patients (26 subjected to internal stabilization with screws), was 47 months (range 24–104 months).

All patients received perioperative antibiotic prophylaxis, were placed in a supine position, and applied a tourniquet (320 mm Hg). An anterior approach centered over the ankle joint was used to create the ankle fusion. Ilizarov apparatus (group 1) or cannulated screws (group 2) were used to achieve compression at the ankle joint. Ilizarov apparatus for ankle arthrodesis consisted of proximal ring fixed to the tibia and fibula with three Kirschner wires, distal ring fixed to the tibia and fibula with two Kirschner wires, and U-shaped foot ring fixed to the calcaneus with two Kirschner wires with olives and to distal part of metatarsal bones with one Kirschner wire with olives. All patients from groups 1 and 2 were operated on by the same three surgeons. Patients from group 1 (subjected to Ilizarov stabilization) started bearing weight on their operated legs on the first postoperative day. Minimum wear time of Ilizarov fixator was 9 weeks. After removal of the device, patients wore walker boots for at least 6 weeks. Subjects from group 2 did not bear weight on their operated legs for at least 6 weeks, and after removal of the cast, continued protected progressive weight bearing in a controlled ankle motion (CAM)-walker for another 6 weeks. Usually, transition to a normal shoe wear took place 3 months after the arthrodesis [26].

The list of outcome measures included (1) balance(path of the center of gravity and the area of the center of gravity) and (2) distribution of lower limb loads, both analyzed separately for patients subjected to external stabilization (group 1) and individuals after internal stabilization with screws(group 2).

All parameters mentioned above were extracted from preoperative medical records and from the histories of postoperative control visits.

**Fig. 1:**
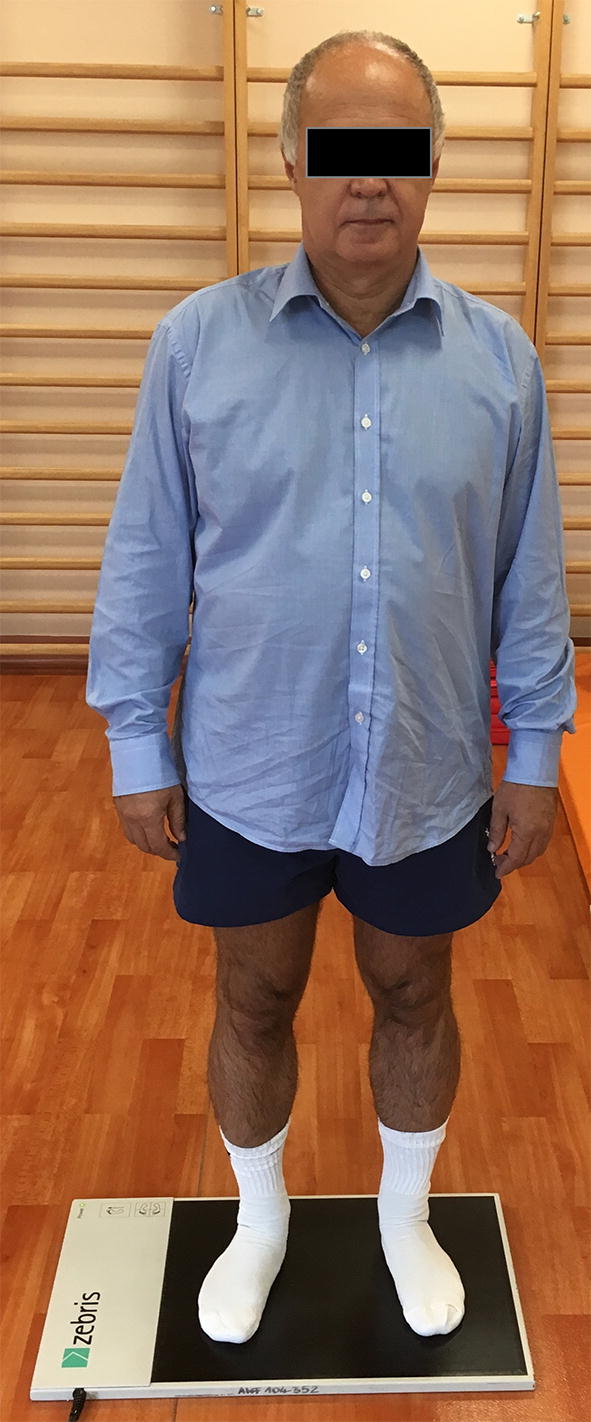
Zebris pedobarographic platform

Balance and distribution of lower limb loads were determined with Zebris pedobarographic platform (Fig. 1) [9–11]. Pedobarograpic platform allows for repeatable and very reliable measurements of static and kinetic parameters of the locomotor system [9–11, 31–33]. It is widely used in the assessment of the course of treatment and treatment results by orthopaedists and physiotherapists [9–11, 31–33]. The platform (320 mm × 470 mm) was equipped with 1504 sensors and connected via a USB cable to a computer installed with FootPrint software. The software was used for processing and recording of pedobarographic parameters that were later subjected to statistical analysis. Patients were examined barefoot, with their eyes open. Before calibration of the platform, each subject was familiarized with the testing procedure [9–11]. Each pedobarographic examination lasted for 90 s and was carried out in a two-legged stance. Each parameter was measured on triplicate and mean value from three measurements was subjected to further analysis. Inside the calculated ellipse, is 95% of measurement data of location of vertical projection of center of gravity [9–11].

The length of vertical projection of the center of gravity (COG) path (the length of the center of gravity line created during the measurement) was expressed in centimeters (cm) and the area of COG (the surface area of the position of the center of gravity created during the measurement) in square centimeters (cm^2^). Distribution of loads between the operated and non-operated limb was expressed in percent [9–11].

Statistical significance of intergroup and intragroup differences in the study variables was examined with Student t-test. The Kolmogorov–Smirnov test was performed to assess normal distribution. All calculations were carried out with Statistica 10 software, with the threshold of statistical significance set at α = 0.05.

## Results

No statistically significant differences were found in demographic characteristics of the study subjects (Table 1).

**Table 1:** Patient demographics/characteristics

Variable	Group 1—Ilizarov external fixator (N = 21)	Group 2—internal stabilization (N = 26)
Age	44 (17–65)	47 (17–67)
Sex	14 (66.6%) male	17 (65.4%) male
follow-up (months)	45 (24–108)	47 (24–104)
Disease diagnosis		
Primary OA	2 (9.5%)	3 (11.5%)
Secondary OA		
Post-traumatic	10 (47.6%)	15 (57.7%)
Rheumatoid	0 (0%)	1 (3.8%)
Congenital	4 (19%)	3 (11.5%)
Neuropathic	5 (23.8%)	4 (15.4%)

Mean loads of the operated and non-operated leg in the Ilizarov stabilization group were 48.8% (SD = 5.3%) and 51.2% (SD = 5.1%), respectively; this intragroup difference was not statistically significant (p = 0.065). Mean loads in the internal stabilization group were 48.4% (SD = 5.9%) and 51.6% (SD = 5.7%) for the operated and non-operated leg, respectively; also this intragroup difference did not reach the threshold of statistical significance (p = 0.069). The study groups did not differ significantly in terms of either the operated or non-operated limb loads (p = 0.059 and p = 0.061, respectively). Mean length of COG path was 137.9 cm (SD = 22.1 cm) for group 1 and 134 cm (SD = 23.4 cm) for group 2; also this intergroup difference was not significant (p = 0.075). Mean COG area was 7.41 cm^2^ (SD = 3.5 cm^2^) for group 1 and 6.16 cm^2^ (SD = 2.9 cm^2^) for group 2; this difference turned out to be statistically significant (p = 0.042) (Table 2).

**Table 2:** Results of pedobarographic tests

Variable	Group 1—Ilizarov external fixator (N = 21)	Group 2—internal stabilization (N = 26)
% of load distribution of the healthy limb	51.2 (SD 5.1)	51.6 (SD 5.7)
% of load distribution of the operated limb	48.8 (SD 5.3)	48.4 (SD 5.9)
Average path length of the center of gravity (cm)	137.9 (SD 22.1)	134 (SD 23.4)
Average area of the center of gravity (cm^2^)	7.41* (SD 3.5)	6.16* (SD 2.9)

* Statistical difference between the group (p < 0.05)

## Discussion

Identification of a stabilization technique that can restore normal biomechanics of the lower limb after ankle arthrodesis is a key component of preoperative planning process.

In this study, we verified if the technique used to stabilize ankle arthrodesis exerts significant effects on (1) body balance and (2) distribution of lower limb loads. To the best of our knowledge, none of the previous studies analyzed these parameters in patients subjected to ankle arthrodesis with various stabilization techniques.

Patients with lower limb deformities have impaired gait biomechanics [41]. Lower limb alignment provides symmetrical distribution of loads between both extremities [11, 40, 42]. According to Rongies et al. patients who experienced lesser pain presented with better balance [32]. Axial correction and lower limb alignment contribute to normalization and symmetry of gait parameters [31], as well as to equal distribution of loads between both extremities [10, 11], but do not provide complete restoration of balance [10]. Gladish et al., studied center of pressure profiles in unilateral compared to bilateral end-stage ankle osteoarthritis patients. They suggested that center of pressure is a better measure of postural strategy while center of mass measures may be more representative of postural steadiness [43]. However, many authors use the COG measurement in assessing the statics of musculoskeletal biomechanics [9–11, 31–33].

Morasiewicz et al. compared balance and distribution of lower limb loads in patients subjected to detorsional corticotomies with the Ilizarov method and in individuals who underwent non-detorsional corticotomies [9]. In another study, the same group analyzed load distributions and balance in patients subjected to corticotomies with the Ilizarov method and in healthy controls [10]. Morasiewicz et al. also compared balance and distribution of lower limb loads in patients in patients before and after Ilizarov corticotomies [11]. Our hereby presented findings and the results of the three studies mentioned above [9–11] are quite consistent, which suggests that pedobarographic measurements are likely reproducible.

In both study groups, ankle fusion turned out to be sufficient to normalize the distribution of lower limb loads at a similar level as in healthy controls [10]. The observation that patients from group 1 and 2 presented with longer COG paths and larger COG areas than previously examined healthy controls [10] may be explained by two mechanisms. First, established compensatory mechanisms of ankle deformity might impair patients’ balance after the arthrodesis procedure as well. Second, worse balance might be associated with some deficits of joint mobility and muscle strength.

Balance in patients from both study groups turned out to be worse than in healthy volunteers [10], which might reflect a post-arthrodesis disruption of lower extremity biomechanics. We do not know how balance was before the surgeries for those patients. But we think that balance was very disturbed due to severe ankle arthritis. Braito et al. demonstrated that patients after ankle arthrodesis presented with a significant gait asymmetry and reduced range of motion [44]. In turn, Wu et al. showed that sagittal plane motion of the hindfoot after this procedure was significantly decreased as compared to healthy subjects; kinematic data documented by these authors corresponded to a generalized stiffness of the hindfoot [45]. On the other hand, Tenenbaum et al. reported that multiple gait parameters were significantly better after ankle arthrodesis than prior to this procedure [46]. In our present study, balance of patients after ankle arthrodesis turned out to be slightly better than in a previously examined subjects who underwent corticotomies with the Ilizarov method [9, 10].

In this study we evaluated two balance parameters (path and area of COG). Path of COG was longer in Ilizarov group than in internal fixation group, but this was not statistically significant. Only area of COG was statistically significant different between groups. It’s hard to explain why only one parameter being different (area of COG), versus both of them, represent in terms of balance. Perhaps this is due to the small size of research groups.

One potential limitation of this study may be the lack of preoperative data on balance and distribution of lower limb loads. This was caused by the fact that only a small number of patients subjected to ankle arthrodesis have been examined with a pedobarographic platform both prior to and after the surgery. During the course of further research, we plan to evaluate pre- and postoperative balance and distribution of lower limb loads in a larger series of patients qualified for ankle arthrodesis. The fact that all surgeries were performed by the same team of three operators using standardized technique should be considered a strength of this study. The same refers to the fact that the same protocol of postoperative management has been used in all the study subjects.

## Conclusions

Ankle arthrodesis with Ilizarov fixation provides worse balance than the same procedure with internal stabilization.

Despite correction of ankle deformity, musculoskeletal biomechanics still remains impaired.

Although either Ilizarov fixation or internal stabilization of ankle arthrodesis result in normalization of lower limb loads, none of these procedures improve patients’ balance.
